# Association of State-Level Tax Policy and Infant Mortality in the United States, 1996-2019

**DOI:** 10.1001/jamanetworkopen.2023.9646

**Published:** 2023-04-24

**Authors:** Jean A. Junior, Lois K. Lee, Eric W. Fleegler, Michael C. Monuteaux, Michelle L. Niescierenko, Amanda M. Stewart

**Affiliations:** 1 Division of Emergency Medicine, Boston Children’s Hospital, Boston, Massachusetts; 2 Department of Pediatrics, Harvard Medical School, Boston, Massachusetts

## Abstract

**Importance:**

Infant mortality in the United States is highest among peer nations; it is also inequitable, with the highest rates among Black infants. The association between tax policy and infant mortality is not well understood.

**Objective:**

To examine the association between state-level tax policy and state-level infant mortality in the US.

**Design, Setting, and Participants:**

This state-level, population-based cross-sectional study investigated the association between tax policy and infant mortality in the US from 1996 through 2019. All US infant births and deaths were included, with data obtained from the National Center for Health Statistics. Data were analyzed from November 28, 2021, to July 9, 2022.

**Exposures:**

State-level tax policy was operationalized as tax revenue per capita and tax progressivity. The Suits index was used to measure tax progressivity, with higher progressivity indicating increased tax rates for wealthier individuals.

**Main Outcomes and Measures:**

The association between tax policy and infant mortality rates was analyzed using a multivariable, negative binomial, generalized estimating equations model. Since 6 years of tax progressivity data were available (1995, 2002, 2009, 2012, 2014, and 2018), 300 state-years were included. Adjusted incidence rate ratios (aIRRs) were calculated controlling for year, state-level demographic variables, federal transfer revenue, and other revenue. Secondary analyses were conducted for racial and ethnic subgroups.

**Results:**

There were 148 336 infant deaths in the US from 1996 through 2019, including 27 861 Hispanic infants, 1882 non-Hispanic American Indian or Alaska Native infants, 5792 non-Hispanic Asian or Pacific Islander infants, 41 560 non-Hispanic Black infants, and 68 666 non-Hispanic White infants. The overall infant mortality rate was 6.29 deaths per 1000 live births. Each $1000 increase in tax revenue per capita was associated with a 2.6% decrease in the infant mortality rate (aIRR, 0.97; 95% CI, 0.95-0.99). An increase of 0.10 in the Suits index (ie, increased tax progressivity) was associated with a 4.6% decrease in the infant mortality rate (aIRR, 0.95; 95% CI, 0.91-0.99). Increased tax progressivity was associated with decreased non-Hispanic White infant mortality (aIRR, 0.95; 95% CI, 0.91-0.99), and increased tax revenue was associated with increased non-Hispanic Black infant mortality (aIRR, 1.04; 95% CI, 1.01-1.08).

**Conclusions and Relevance:**

In this cross-sectional study, an increase in tax revenue and the Suits index of tax progressivity were both associated with decreased infant mortality. These associations varied by race and ethnicity. Tax policy is an important, modifiable social determinant of health that may influence state-level infant mortality.

## Key Points

**Question:**

Is state-level tax policy associated with infant mortality in the United States?

**Findings:**

In this cross-sectional study of 148 336 infant deaths from 1996 through 2019, an increase in tax revenue and the Suits index of tax progressivity were both statistically significantly associated with decreased infant mortality. These associations varied by race and ethnicity.

**Meaning:**

These findings suggest that tax policy, an important and modifiable social determinant of health, may be one approach for decreasing infant mortality.

## Teaser

This cross-sectional study examines the association between state-level tax policy and state-level infant mortality in the US overall and by race and ethnicity.

## Introduction

Infant mortality is one of the most important indicators of population health.^1^ The United States has had the highest infant mortality rate among wealthy nations for decades,^2^ despite spending more per capita on health care than any other country.^3^ US infant mortality is also inequitable. The rate of infant mortality among non-Hispanic Black individuals is higher than that of any other racial group and is more than twice that among non-Hispanic White individuals.^4^

Social determinants of health, including poverty, inequality, and racism, influence infant mortality via multiple mechanisms.^5,6^ For example, chronic poverty and racism are associated with toxic stress and dysregulated cortisol secretion,^7,8^ increased smoking rates during pregnancy,^9^ and increased risk of prematurity^10^ and sudden unexplained infant death.^8^ In societies with high inequality, pregnant people experiencing relative deprivation may have increased psychosocial stress that contributes to adverse neonatal outcomes.^5^

Tax policy is theorized to be an upstream social determinant that may generate tax revenue and redistribute wealth, thereby mitigating poverty and inequality and improving health outcomes.^11^ Indeed, tax revenues fund myriad government programs^12^ that are associated with poverty mitigation^13,14^ and infant mortality reduction.^15,16,17,18,19^ These include the Earned Income Tax Credit (EITC; a refundable tax credit for low-income workers), Medicaid, evidence-based home visiting programs, and the Supplemental Nutrition Program for Women, Infants, and Children.

Progressive taxes require wealthier individuals to pay higher tax rates than less wealthy individuals while regressive taxes do the opposite.^20^ The EITC makes tax systems overall more progressive. In contrast, sales taxes on necessities, such as food, are generally regressive, because spending on necessities consumes a higher proportion of financial resources for lower-income individuals.^20,21^ Nonfederal taxes are important revenue sources, because states and localities are responsible for most direct domestic program spending.^22^

Prior research on taxes and health outcomes has focused on the EITC^19^ and so-called *sin taxes*, such as tobacco taxes and sweetened beverage taxes.^23^ Research on the overall tax system as a social determinant of health is limited. The objective of this study was to examine the association between state-level tax policy and state-level infant mortality in the US. We hypothesized that increased tax revenue is associated with decreased infant mortality and increased tax progressivity is associated with decreased infant mortality.

## Methods

### Study Design and Data Sources

We conducted a state-level, ecologic, cross-sectional study investigating the association between tax policy and infant mortality in the US from 1996 through 2019. This study was determined not to be human participants research by the Boston Children’s Hospital institutional review board. We followed the Strengthening the Reporting of Observational Studies in Epidemiology (STROBE) reporting guideline.

We obtained counts of births and deaths for infants aged 0 to 364 days from the Centers for Disease Control and Prevention’s National Center for Health Statistics (NCHS).^4,24^ We signed an NCHS data use agreement granting access to the full, period-linked database without suppression of low counts.^24^ We obtained tax revenue data from the US Census Bureau Annual Survey of State and Local Government Finances^25^ and tax progressivity data from the Institute on Taxation and Economic Policy (ITEP).^20,26,27,28,29,30^ The US Census Bureau was also our source of state population counts^31^ and all covariates included in our analyses.^25,31,32,33,34,35,36^ Our sources had no missing data.

### Study Measures

Our primary dependent variable was state-level infant mortality rate per 1000 live births. Our secondary dependent variables were infant mortality rates per 1000 live births for Hispanic infants of all races, non-Hispanic American Indian or Alaska Native infants, non-Hispanic Asian or Pacific Islander infants, non-Hispanic Black infants, and non-Hispanic White infants. We defined each infant’s race and Hispanic origin as their mother’s race and Hispanic origin listed in the NCHS database.^4^

Our first independent variable was tax revenue. For each state, we defined tax revenue as all revenues from statewide and local sources classified by the US Census Bureau as taxes.^25^ Our second independent variable was tax progressivity. ITEP tax progressivity data were available for 6 years during the study period: 1995, 2002, 2009, 2012, 2014, and 2018.^20,26,27,28,29,30^ To our knowledge, ITEP is the only source of state-level effective tax rates combining personal income, property, and sales taxes. These comprehensive tax data were not available at the county or local level. We used ITEP data to calculate a Suits index for each state following methods used in prior studies.^37,38^ The Suits index is a commonly used measure of tax progressivity.^39,40,41^ Its values range from −1 (a maximally regressive tax) to 1 (a maximally progressive tax), with a Suits index of 0 representing a proportionate tax (neither regressive nor progressive).^39^

Our selection process for state-level covariates is described in the eMethods in Supplement 1. Selected covariates were year, federal transfer revenue,^25^ other revenue,^25^ non-Hispanic Black population percentage,^31^ Hispanic population percentage,^31^ median household income,^32^ and percentage of population aged 25 years and older that graduated from high school.^33,34,35,36^ Federal transfer revenue is revenue transferred from the federal government to state governments, which helps compensate for inadequacies in state tax revenues.^22,42,43^ We defined other revenue as all revenue from sources other than federal transfer revenue, state taxes, and local taxes. Other revenue includes fees (eg, state university tuition), fines (eg, traffic violations), and earnings from government investments.^25,44^ State and local nontax revenue sources are generally regressive.^20^ All government revenue and median household income data were inflation-adjusted using US Department of Commerce price indexes and are reported in 2020 US dollars.^45^ Revenue values for each year are reported per capita based on the population of each state for that year. The data set we compiled of independent variables and covariates is available on request.

### Statistical Analysis

We conducted 2 univariate analyses: one for the association between state-level tax revenue per capita and state-level infant mortality and another for the association between state-level tax progressivity and state-level infant mortality. Our primary analysis examined the association between our 2 state-level tax policy variables and state-level infant mortality with a multivariable model adjusting for all covariates. We conducted secondary analyses of the association between state-level tax policy and state-level infant mortality for Hispanic infants of all races, non-Hispanic American Indian or Alaska Native infants, non-Hispanic Asian or Pacific Islander infants, non-Hispanic Black infants, and non-Hispanic White infants.

For each analysis, we used a negative binomial generalized estimating equations model to report incidence rate ratios (IRRs) with 95% CIs. Models used infant death counts as the dependent variable and the log of infant birth counts as the offset (coefficient constrained to 1). The state-year was the unit of analysis. Since each of the 50 states had 6 years of tax progressivity data available, 300 state-years were included. We used a 1-year lag between tax policy and infant mortality. Thus, we used tax policy and covariate data spanning 1995 through 2018 paired with mortality data spanning 1996 through 2019. Whenever possible, we used an unstructured correlation matrix. We used an exchangeable correlation matrix for models that did not converge when an unstructured correlation matrix was used.^46,47^ Robust SEs adjusted for clustering by state.

For our primary and secondary analyses, we used multivariable models adjusting for all covariates, and reported adjusted IRRs (aIRRs). We modeled year as a set of dummy variables, with our initial year of study as the reference. For our primary analysis, we calculated a variance inflation factor (VIF) for each independent variable and covariate to assess for multicollinearity.

We also conducted 2 sensitivity analyses. First, we repeated the primary analysis using 2-year and 3-year lags between tax policy and infant mortality. Second, we repeated the primary analysis using the Kakwani index of tax progressivity.^48^ The Kakwani index is another commonly used progressivity measure ranging from −1 (a maximally regressive tax) to 1 (a maximally progressive tax), with a Kakwani index of 0 representing a proportionate tax. This index can yield different values than the Suits index, depending on the pretax income distribution.^48^

As described in the eMethods in Supplement 1, we conducted post hoc sensitivity analyses in which we modified the primary analysis. First, we used state fixed effects. Second, we controlled for political context (eg, percentage of state legislators who were Democrats). Third, we controlled for the percentage of infant deaths comprised of out-of-state residents. Fourth, we accounted for the 1 included state-year with data deemed unreliable by the NCHS due to the number of infant deaths being less than 20.^49^ This state-year was Vermont in 2019, which had 15 infant deaths. We repeated the primary analysis with Vermont’s 2019 infant deaths increased first to 19, then to 20.

We conducted an exploratory analysis investigating whether the association between tax progressivity and infant mortality is modified by tax revenue. To do this, we added to the primary analysis an interaction term between tax revenue and the Suits index of tax progressivity. We planned all analyses a priori except for the post hoc sensitivity analyses. All tests were 2-tailed and used a significance level of *P* = .05. We conducted analyses using Stata statistical software version 17.0 (StataCorp). Data were analyzed from November 28, 2021, to July 9, 2022.

## Results

### Descriptive Statistics and Univariate Analyses

**Figure 1.**
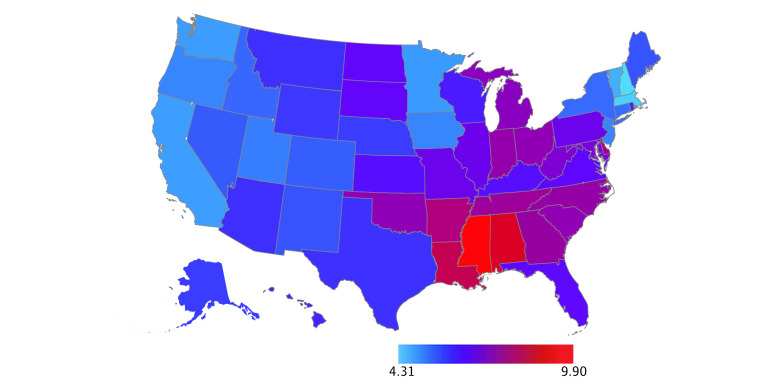
Mean of State-Level Infant Mortality Rates per 1000 Live Births Over the 6 Years Studied Mortality rate data were from 1996, 2003, 2010, 2013, 2015, and 2019 (the 6 years with corresponding tax progressivity data available).

**Figure 2.**
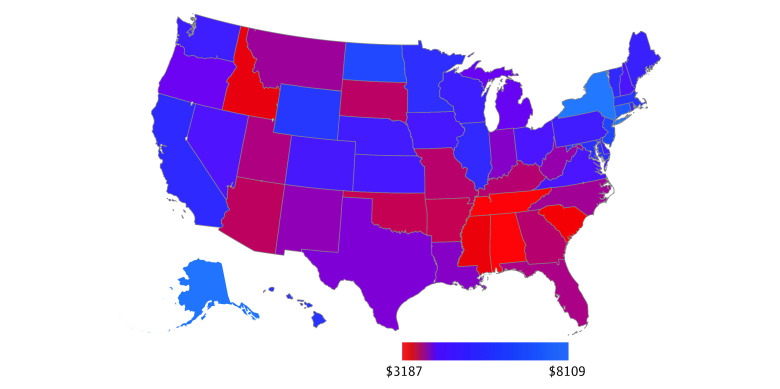
Mean of State-Level Tax Revenue per Capita Over the 6 Years Studied Tax revenue data were from 1995, 2002, 2009, 2012, 2014, and 2018 (the 6 years with corresponding tax progressivity data available).

**Figure 3.**
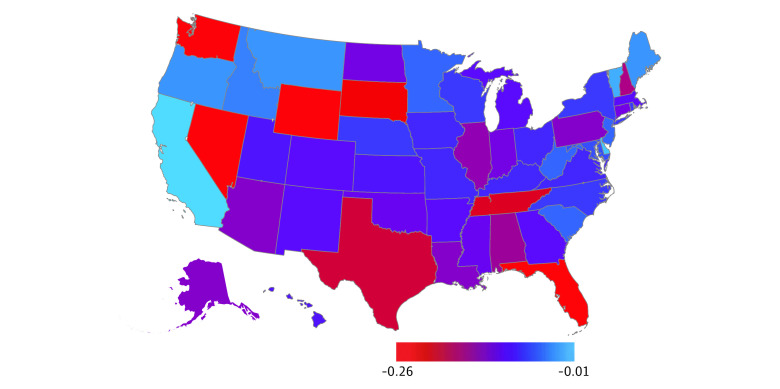
Mean State-Level Suits Indices of Tax Progressivity Over the 6 Years Studied Tax progressivity data were only available for 1995, 2002, 2009, 2012, 2014, and 2018.

For the 6 years with state-level tax progressivity data available, there were 148 336 infant deaths, 23 585 986 live births, and an overall infant mortality rate of 6.29 deaths per 1000 live births (Table 1). By race and ethnicity, there were 27 861 deaths among Hispanic infants, 1882 deaths among non-Hispanic American Indian or Alaska Native infants, 5792 deaths among non-Hispanic Asian or Pacific Islander infants, 41 560 deaths among non-Hispanic Black infants, and 68 666 deaths among non-Hispanic White infants. Comparing all state-years studied, the lowest infant mortality rate was 2.80 deaths per 1000 live births (in Vermont in 2019), and the highest infant mortality rate was 10.93 deaths per 1000 live births (in Mississippi in 1996) (eTable 1 in Supplement 1). When we calculated the mean over the 6 years studied, New Hampshire had the lowest mean state-level infant mortality rate, at 4.31 deaths per 1000 live births, and Mississippi had the highest mean state-level infant mortality rate, at 9.90 deaths per 1000 live births (Figure 1). Across all state-years studied, the median (IQR) tax revenue per capita was $4275 ($3746-$5121) and the mean (SD) Suits Index was −0.11 (0.06) (eTable 1 in Supplement 1). When we calculated means over the 6 years studied, the lowest tax revenue per capita was in Alabama ($3187) and the highest was in New York ($8109) (Figure 2). Regressive taxes were present in 294 out of the 300 state-years studied. When we calculated means over the 6 years studied, all states had regressive taxes (Figure 3). Univariate analyses demonstrated a decreased infant mortality rate for each $1000 increase in tax revenue per capita (IRR, 0.94; 95% CI, 0.90-0.98) and each 0.10-unit increase in the Suits index (IRR, 0.90; 95% CI, 0.84-0.97).

**Table 1.** Infant Deaths, Births, and Mortality Rates by Subgroup^a^

Measure	All infants^b^	Hispanic infants	Non-Hispanic infants			
			American Indian or Alaska Native	Asian or Pacific Islander	Black	White
Deaths	148 336	27 861	1882	5792	41 560	68 666
Births	23 585 986	5 263 373	221 938	1 385 545	3 464 107	13 042 559
Infant mortality rate per 1000 live births	6.29	5.29	8.48	4.18	12.00	5.26

^a^ Mortality rate data were from 1996, 2003, 2010, 2013, 2015, and 2019 (the 6 years with corresponding tax progressivity data available).

^b^ Includes infants whose Hispanic origin is unknown or not stated. Thus, the number of births in this column is greater than the sum of the births in the other columns, and the number of deaths in this column is greater than the sum of the deaths in the other columns.

### Primary Analysis

In the multivariable model examining the association between tax policy and infant mortality, each $1000 increase in tax revenue per capita was associated with a 2.6% decrease in the infant mortality rate (aIRR, 0.97; 95% CI, 0.95-0.99) (Table 2). An increase of 0.10 in the Suits index (ie, increased tax progressivity) was associated with a 4.6% decrease in the infant mortality rate (aIRR, 0.95; 95% CI, 0.91-0.99). There was no evidence of multicollinearity (VIFs of all independent variables and covariates were <5) (eTable 2 in Supplement 1).

**Table 2.** Multivariable Model of the Association Between Tax Policy and Infant Mortality

Variable	Infant mortality, aIRR (95% CI)			
	All infants	Hispanic infants	Non-Hispanic infants^a^	
			Black	White
Independent variables				
Tax revenue per capita^b^	0.97 (0.95-0.99)	1.01 (0.98-1.04)	1.04 (1.01-1.08)	0.98 (0.95-1.00)
Suits index of tax progressivity^c^	0.95 (0.91-0.99)	1.03 (0.96-1.10)	0.99 (0.93-1.07)	0.95 (0.91-0.99)
Covariates^d^				
Federal transfer revenue per capita^b^	0.97 (0.93-1.00)	0.98 (0.90-1.08)	0.89 (0.81-0.97)	0.96 (0.92-0.99)
Other revenue per capita^b^	1.02 (1.01-1.03)	1.00 (0.95-1.05)	0.99 (0.97-1.02)	1.02 (1.00-1.03)
Non-Hispanic Black population^e^	1.11 (1.08-1.14)	1.02 (0.95-1.09)	1.09 (1.03-1.15)	1.00 (0.97-1.03)
Hispanic population^e^	0.95 (0.92-0.98)	0.97 (0.92-1.03)	1.00 (0.95-1.04)	0.95 (0.92-0.98)
High school graduation rate^f^	0.99 (0.95-1.02)	1.06 (0.95-1.18)	1.07 (0.98-1.17)	0.98 (0.94-1.02)
Median household income^g^	0.95 (0.92-0.98)	0.97 (0.92-1.01)	0.91 (0.86-0.96)	0.90 (0.87-0.94)

Abbreviation: aIRR, adjusted incidence rate ratio.

^a^ Given the high proportion of state-years with data deemed unreliable by the NCHS (eTable 2 in Supplement 1), results are not shown for non-Hispanic American Indian and Alaska Native and non-Hispanic Asian and Pacific Islander subgroups. However, these subgroup analysis results are available on request.

^b^ For all revenue variables, the aIRR shown applies to a $1000 increase in revenue.

^c^ For the Suits index of tax progressivity, the aIRR shown applies to a 0.10-unit increase in the index.

^d^ Year dummy variables are not shown.

^e^ For non-Hispanic Black population, the aIRR shown applies to a 10% increase in percentage of the population that is non-Hispanic Black. For Hispanic population, the aIRR shown applies to a 10% increase in percentage of the population that is Hispanic.

^f^ For high school graduation rate, the aIRR shown applies to a 5% increase in graduation rate.

^g^ For median household income, the aIRR shown applies to a $10 000 increase in median household income.

### Secondary Analyses by Race and Ethnicity

For non-Hispanic Black infants, each $1000 increase in tax revenue per capita was associated with a 4.1% increase in infant mortality rate (aIRR, 1.04; 95% CI, 1.01-1.08) (Table 2). For non-Hispanic White infants, an increase of 0.10 in the Suits index was associated with a 4.8% decrease in the infant mortality rate (aIRR, 0.95; 95% CI, 0.91-0.99). There was no statistically significant association between tax policy and infant mortality for other racial and ethnic subgroups. Except for non-Hispanic White infants, all subgroups had fewer than 20 infant deaths for more than 30% of state-years studied (eTable 3 in Supplement 1).

### Sensitivity Analyses

In the sensitivity analyses using 2-year and 3-year lags, there were no statistically significant associations between infant mortality rate and either tax revenue per capita or tax progressivity (eTable 4 in Supplement 1). When the Kakwani index of tax progressivity was used, the negative association between tax progressivity and infant mortality rate did not remain statistically significant (aIRR, 0.95; 95% CI, 0.90-1.00). The negative association between tax progressivity and infant mortality remained statistically significant when we increased Vermont’s 2019 infant deaths from 15 to 19, but not when we increased Vermont’s 2019 infant deaths from 15 to 20. Otherwise, the associations between tax policy and infant mortality found in our primary analysis remained robust in all sensitivity analyses (eTable 4 and 5 in Supplement 1).

### Exploratory Analyses

The interaction term between tax revenue and the Suits index was significant (aIRR, 0.97; 95% CI, 0.96-0.99). As tax revenue increased, the association between the Suits index of tax progressivity and infant mortality rate became more negative (ie, increased progressivity was associated with larger decreases in infant mortality) (eTable 6 in Supplement 1). For tax revenues per capita of $5550 and greater, there was a statistically significant negative association between the Suits index and infant mortality (eTable 6 in Supplement 1).

## Discussion

In this state-level cross-sectional study, increases in tax revenue and the Suits index of tax progressivity were both associated with decreased infant mortality, controlling for multiple covariates. These associations varied by race and ethnicity.

Excluding research on the EITC and sin taxes, there are limited studies on the association between tax policy and mortality in the US.^50,51,52,53^ Three of these studies included infant mortality as an outcome, and they reported mixed results.^50,51,52^ To our knowledge, our study makes 2 novel contributions. First is our use of a well-accepted tax progressivity measure that accounts for the entire income spectrum. This is important, because previous studies have demonstrated decreased mortality rates for each incremental increase in education^54^ and income^55^ in the US. Second is our examination of the association between tax policy and mortality for different racial and ethnic subgroups.

Many socioeconomic policies beyond taxes likely contribute to infant mortality, including minimum wage laws,^56^ ratio of government social spending to health spending,^57^ and access to paid family leave.^58^ Furthermore, structural racism is intertwined with infant mortality^59,60,61^ and tax policy. Slave owner resistance to taxes on enslaved individuals and post–Civil War opposition to raising tax revenue that could benefit non-Hispanic Black individuals have contributed to enduring regressive taxes and low tax revenues in some states.^37,43^

Structural racism may help explain our finding that increased tax revenue was associated with increased non-Hispanic Black infant mortality. Numerous studies document racial inequities in safety-net program access, service quality, and outcomes.^62,63,64,65,66^ Thus, in some cases, low-income non-Hispanic Black individuals may lose more from paying taxes than they gain from tax-funded government services. This may contribute to higher poverty rates among non-Hispanic Black individuals, worse inequities between non-Hispanic Black and non-Hispanic White individuals, and worse non-Hispanic Black infant mortality rates. Fortunately, as demonstrated by the EITC, policies and programs can be designed to both decrease overall infant mortality^19^ and help promote racial equity.^67,68,69^ The lack of association between tax policy and Hispanic infant mortality may be due to relatively limited numbers of infant deaths, racial and ethnic misclassification on death certificates,^70^ and heterogeneous experiences of racism among Hispanic individuals.

Associations between tax policy and infant mortality may wane after a year. This could be due to temporal changes in tax and other policies. However, our sensitivity analyses with 2- and 3-year lags excluded 50 state-years, because infant mortality data was not yet available for 2020 onward. Thus, these analyses had less power to detect possible associations between tax policy and infant mortality.

Our sensitivity analyses suggest that, compared with tax revenue, tax progressivity may have a less robust association with infant mortality. However, there was limited variation in tax progressivity among states, as illustrated by the fact that 294 of the 300 state-years studied had regressive taxes. Thus, there may have been inadequate variation in the Kakwani index of tax progressivity to detect an association between this index and infant mortality. Because Vermont had relatively high tax revenue and progressivity, increasing its 2019 infant deaths to 20 attenuated our primary analysis results.

Our exploratory analyses suggest that there may be a minimum level of tax revenue necessary for a negative association between tax progressivity and infant mortality. If total tax revenue is inadequate, even a highly progressive tax system would not likely translate into meaningfully increased government funding for programs associated with decreased infant mortality. Thus, increasing tax progressivity without also increasing tax revenue may not contribute to decreased infant mortality.

Tax policy deserves greater attention from researchers and advocates as a means of improving health outcomes. First, tax revenue is potentially important for numerous health-related funding priorities. Second, as an underlying social determinant of health, tax policy could potentially affect numerous health outcomes. Finally, increasing tax revenue and progressivity is economically feasible. Hundreds of billions of dollars of taxes owed remain uncollected each year.^71,72^ There is strong evidence that tax cuts for the wealthy do not affect economic growth.^73^ Additionally, using tax revenue to reduce child poverty is estimated to save money in the long-term by increasing adult productivity and reducing crime and health care costs.^62^

### Limitations

This study has limitations. There is the potential for measurement error in the NCHS, ITEP, and US Census Bureau data used. We were not able to include every year during the study period, as only 6 years of comprehensive tax progressivity data were available. Our primary and secondary analyses do not account for potential nonlinear threshold effects, ie, associations between tax policy and infant mortality that exist only above or below certain levels of tax revenue or tax progressivity. Secondary analyses may be unreliable, given small numbers of infant deaths. Although using 1-, 2-, and 3-year lags seemed reasonable based on prior studies,^15,52,56^ the time frame in which tax policy may influence health outcomes is unclear. Given that this is an ecological study, there may be unmeasured individual- or population-level confounders. Thus, conclusions about causality cannot be drawn.

## Conclusions

In this cross-sectional study of state-level tax policy and infant mortality, we found that increases in tax revenue and the Suits index of tax progressivity were both associated with decreased infant mortality. These associations varied by race and ethnicity. Influencing tax systems may be one approach in a multipronged strategy to decrease rates of, and inequities in, infant mortality. Tax policy is a potentially important, modifiable social determinant of health that deserves greater attention from researchers, advocates, and policy makers.

## Notes

Supplement 1. **eMethods.** Covariate Selection and Details of Post Hoc Sensitivity Analyses **eTable 1.** Characteristics of the 300 State-Years Studied **eTable 2.** Variance Inflation Factor for Each Independent Variable and Covariate **eTable 3.** State-Years With Fewer Than 20 Infant Deaths **eTable 4.** A Priori Planned Sensitivity Analyses of the Association Between Tax Policy and Infant Mortality **eTable 5.** Post Hoc Sensitivity Analyses of the Association Between Tax Policy and Infant Mortality **eTable 6.** Association Between the Suits Index Of Tax Progressivity and Infant Mortality at Different Levels of Tax Revenue Per Capita
eReferences

Supplement 2.
Data Sharing Statement
