# Supplementary material for: Association of State-Level Tax Policy and Infant Mortality in the United States, 1996-2019
Source: JAMA Netw Open. 2023 Apr 24;6(4):e239646. doi: 10.1001/jamanetworkopen.2023.9646 (PMC10126872; doi:10.1001/jamanetworkopen.2023.9646)
Supplement: Supplement 1. — eMethods. Covariate Selection and Details of Post Hoc Sensitivity Analyses eTable 1. Characteristics of the 300 State-Years Studied eTable 2. Variance Inflation Factor for Each Independent Variable and Covariate eTable 3. State-Years With Fewer Than 20 Infant Deaths eTable 4. A Priori Planned Sensitivity Analyses of the Association Between Tax Policy and Infant Mortality eTable 5. Post Hoc Sensitivity Analyses of the Association Between Tax Policy and Infant Mortality eTable 6. Association Between the Suits Index Of Tax Progressivity and Infant Mortality at Different Levels of Tax Revenue Per Capita eReferences [file jamanetwopen-e239646-s001.pdf]

Supplemental Online Content

Junior JA, Lee LK, Fleegler EW, Monuteaux MC, Niescierenko ML, Stewart AM. Association of state-level tax policy and infant mortality in the United States, 1996-2019. *JAMA Netw Open*. 2023;6(4):e239646. doi:10.1001/jamanetworkopen.2023.9646

eMethods. Covariate Selection and Details of Post Hoc Sensitivity Analyses
eTable 1. Characteristics of the 300 State-Years Studied
eTable 2. Variance Inflation Factor for Each Independent Variable and Covariate
eTable 3. State-Years With Fewer Than 20 Infant Deaths
eTable 4. A Priori Planned Sensitivity Analyses of the Association Between Tax Policy and Infant Mortality
eTable 5. Post Hoc Sensitivity Analyses of the Association Between Tax Policy and Infant Mortality
eTable 6. Association Between the Suits Index Of Tax Progressivity and Infant Mortality at Different Levels of Tax Revenue Per Capita
eReferences

This supplemental material has been provided by the authors to give readers additional information about their work.

© 2023 Junior JA et al. *JAMA Network Open.*

eMethods. Covariate Selection and Details of Post Hoc Sensitivity Analyses

Covariate selection

We reviewed existing studies on the association between population-level socioeconomic conditions/policies and infant mortality. We identified 28 studies that discussed potential state-level covariates relevant to our investigation.[1,2,11–20,3,21–28,4–10] We considered each of these covariates for inclusion. For our analyses, we aimed for a ratio of at least 10-15 observations for each predictor (including independent variables, year dummy variables, and covariates).[29,30] To limit the number of predictors included, we only included covariates that we thought would meet the following 5 criteria, the first 3 of which define a confounder[31,32]:

1. Associated with an independent variable investigated
2. Associated with the dependent variable investigated
3. Not in the causal pathway between the independent and dependent variables investigated
4. State-level (rather than local-, community-, or individual-level)
5. Relatively commonly used among other studies on the association between population-level socioeconomic conditions/policies and infant mortality

Only state-level covariates were included given the population-level focus of our ecologic study. All covariates were selected a priori.

We controlled for all non-tax revenue to allow for examination of the association between tax revenue and infant mortality independent of all other revenue sources. Non-tax revenue can be categorized as federal transfer revenue and other revenue. Since non-tax revenue can be used instead of taxes to fund government programs, increased non-tax revenue seemed likely to be associated with reduced need for tax revenue. Federal transfer revenue has been associated with decreased infant mortality.[13] Since other revenue is comprised, in part, of fines and fees that can disproportionately burden non-Hispanic Blacks,[33–35] we thought that other revenue may contribute to racial disparities in poverty and thus in infant mortality. For this reason, we included federal transfer revenue and other revenue as two separate covariates.

Since infant mortality rates vary by racial and ethnic group,[36] population percentage that is non-Hispanic Black and population percentage that is Hispanic both seemed likely to be associated with infant mortality rate. Increased non-Hispanic Black population percentage and increased Hispanic population percentage have both been associated with decreased tax progressivity.[37,38] Thus, we included both as covariates.

Higher education and income have both been associated with decreased infant mortality.[11,18,27] Increased education (eg, high school completion) has been associated with increased support for conservative economic policies[39] and decreased support for redistribution from the rich to the poor.[40] Increased median household income provides a larger tax base, and thus potentially more tax revenue. Thus, we included median household income, and population percentage ages 25 and older that graduated from high school, as covariates.

We excluded potential covariates that seemed unlikely to satisfy the 5 aforementioned criteria. We excluded measures of state-level inequality, since inequality alone has been found to lack an association with more progressive taxation.[41] Additionally, we did not include measure of inequality since reduced inequality was on our theorized causal pathway between increased tax progressivity and reduced infant mortality. We excluded economic measures, such as poverty rate, unemployment rates, and gross domestic product as covariates, as these are likely to be collinear with median household income. Health and social services at least partially funded by government revenue (eg, Medicaid, available health center, and the Supplemental Nutrition Program for Women, Infants, and Children) were on our theorized causal pathway between increased tax revenue and reduced infant mortality. Thus, we excluded these services as covariates. We excluded the actual percentages of non-Hispanic Black and Hispanic births in our infant mortality data as covariates, since they were not population-level variables. Additionally, only non-Hispanic Black and Hispanic overall population percentages (rather than infant population percentages) have been associated with state-level tax progressivity based on the literature we identified. Though we considered political climate (eg, party in control of the state legislature) as a covariate, this was not commonly controlled for in the studies we reviewed. Additionally, the studies we reviewed that did control for party in control of the state legislature did not find it to be statistically significantly associated with infant mortality.[4,7] Thus, we did not include political climate as a covariate.

Post hoc sensitivity analyses

After completing our planned analyses, we decided post hoc to conduct additional sensitivity analyses to test the robustness of our primary analysis results.

First, we repeated the primary analysis using state fixed effects, rather than a generalized estimating equations model with clustering by state. An unconditional fixed effects model would include dummy variables to represent each state (ie, 50 – 1 = 49 dummy variables). This model has the advantage of controlling for potential time-invariant unmeasured state-level confounders.[42,43] However, this model has the disadvantage of the incidental parameters problem.[44,45] Additionally, given that there were only 300 observations for our study, including 49 state dummy variables in addition to our 2 independent variables, 5 year dummy variables, and 6 covariates would result in an observations to predictors ratio of 4.8:1, well below the commonly recommended 10:1 ratio.[30] For these reasons, conditional fixed effects models have been deemed preferable to unconditional fixed effects models for studies like ours with > 20 clusters.[44] However, conditional fixed effects models do not control for all predictors and have thus been critiqued as not being true fixed effects.[44] Given these issues with fixed effects models, generalized estimating equations models are among the most commonly used for analyzing clustered data.[44,46] Nevertheless, we tested whether or not our primary analysis was robust to the use of conditional fixed effects.

Second, we repeated the primary analysis three times, each time using a different state-level variable to control for political climate. The first variable used was the percentage of state legislators who were Democrat. The sensitivity analysis using

© 2023 Junior JA et al. *JAMA Network Open.*

this variable excludes data from Nebraska, as Nebraska has a non-partisan legislature. The second variable was binary, with 1 representing a state legislature in which the majority of legislators were Democrat, and 0 representing a state legislature in which the majority of legislators were Republican. For Nebraska and the one state-year (Ohio in 1996) in which exactly 50% of state legislators were Democrat, if the Governor was Democrat, the state-year was coded as Democrat, and if the Governor was Republican, the state-year was coded as Republican. For these variables, we used data from the Council of State Governments Book of States.[47] Whenever possible, we used a 1-year lag between these variables and infant mortality. If data were unavailable for a 1-year lag, we used a 2-year lag. The third political climate variable was the percentage of United States presidential election votes cast for the Democratic Party candidate. For this variable, we obtained data from the Massachusetts Institute of Technology (MIT) Election Data and Science Lab for the most recent election prior to each year of infant mortality data.[48]

Our third sensitivity analysis repeated the primary analysis controlling for the percentage of infant deaths comprised of out-of-state residents. 96% of the 148 336 infant deaths included in our analysis were infants who died in the same state where they were documented as residing at the time of death. The other 4% died in a different state. These non-resident infant deaths are included in the official, publicly available, state-level infant death counts produced by the Centers for Disease Control and Prevention's National Center for Health Statistics (NCHS).[49] People who change residence while pregnant tend to be younger, of lower parity, and of lower socioeconomic status than women who do not move while pregnant.[50] Thus, to align with official statistics and avoid bias from exclusion of a sub-population at potentially higher risk of infant mortality, we included non-resident infant births and deaths in our primary analysis. However, some states, such as those with large children's hospitals to which critically ill infants are frequently transferred from out-of-state, may attract a relatively high number of non-resident infants at high risk of death. To attempt to account for states' propensities to attract high-risk non-resident infants, we did a sensitivity analysis controlling for the percentage of infant deaths comprised of out-of-state residents.

When infant deaths for a given geography and timeframe are < 20, the NCHS deems the number of these deaths to be unreliable, because the number is too small to meet NCHS standards of reliability and precision.[51] Thus, our fourth sensitivity analysis accounted for the one state-year with < 20 infant deaths. This state-year was Vermont in 2019, which had 15 infant deaths. We repeated the primary analysis first with Vermont's 2019 infant deaths increased from 15 to 19, then with Vermont's 2019 infant deaths increased from 15 to 20.

© 2023 Junior JA et al. *JAMA Network Open.*

eTable 1. Characteristics of the 300 State-Years Studied

Characteristic	Median (IQR)	Mean (SD)	Minimum	Maximum
Infant mortality rate per 1000	6.37 (5.27 to 7.27)	6.38 (1.41)	2.80	10.93
Tax revenue per capita, $[a]	4275 (3746 to 5121)	4657 (1425)	2624	13 200
Suits index of tax progressivity	-0.10 (-0.15 to -0.07)	-0.11 (0.06)	-0.30	0.06
Kakwani index of tax progressivity	-0.08 (-0.12 to -0.05)	-0.09 (0.06)	-0.24	0.03
Federal transfer revenue per capita, $	1953 (1604 to 2365)	2081 (770)	736	6429
Other revenue per capita, $[b]	3236 (2417 to 4364)	3478 (1858)	-2175	16 187
Black population, %	7.31 (3.12 to 15.07)	10.56 (9.46)	0.30	37.98
Hispanic population, %	6.47 (3.39 to 12.10)	9.94 (9.82)	0.49	49.32
High school graduation rate, %	87.60 (84.45 to 90.30)	86.93 (4.21)	72.70	93.90
Median household income, $	59 100 (52 618 to 65 396)	59 718 (9917)	38 248	88 664

Abbreviations: IRQ, interquartile range; SD, standard deviation.
[a] All revenue and income values are in 2020 US dollars.
[b] Other revenue was negative when government investments lost substantial amounts of money.

© 2023 Junior JA et al. *JAMA Network Open.*

eTable 2. Variance Inflation Factor[a] for Each Independent Variable and Covariate

	Variance inflation factor
Year	2.54
Tax revenue per capita	2.35
Suits index of tax progressivity	1.06
Federal transfer revenue per capita	1.91
Other revenue per capita	1.33
Black population %	1.83
Hispanic population %	1.63
High school graduation rate	4.01
Median household income	2.56

[a] Variance inflation factors < 5 indicate that there was not multicollinearity.

© 2023 Junior JA et al. *JAMA Network Open.*

eTable 3. State-Years With Fewer Than 20 Infant Deaths

	All infants N (%)	Hispanic infants N (%)	Non-Hispanic infants			
			American Indian or Alaska Native N (%)	Asian or Pacific Islander N (%)	Black N (%)	White N (%)
State years in which there were < 20 infant deaths[a]	1 (0.33)	108 (36.00)	272 (90.67)	222 (74.00)	92 (30.67)	7 (2.33)

Abbreviations: N, number of infants

[a] When infant deaths for a given geography and timeframe are < 20, the NCHS deems the number of these deaths to be unreliable, because the number is too small to meet NCHS standards of reliability and precision.[51]

© 2023 Junior JA et al. *JAMA Network Open.*

eTable 4. A Priori Planned Sensitivity Analyses of the Association Between Tax Policy and Infant Mortality

	Infant mortality aIRR (95% CI)		
	**2-year lag (N = 250)**	**3-year lag (N = 250)**	**Kakwani index (N = 300)**
**Independent variables**			
Tax revenue per capita[a]	1.00 (0.99-1.02)	0.99 (0.96-1.02)	0.97 (0.95-0.99)
Suits index of tax progressivity[b]	0.97 (0.94-1.01)	0.98 (0.94-1.02)	N/A
Kakwani index of tax progressivity[b]	N/A	N/A	0.95 (0.90-1.00)
**Covariates[c]**			
Federal transfer revenue per capita[a]	1.00 (0.97-1.03)	1.02 (0.97-1.07)	0.97 (0.93-1.00)
Other revenue per capita[a]	1.00 (0.99-1.02)	0.99 (0.98-1.00)	1.02 (1.00-1.03)
Non-Hispanic Black population[d]	1.13 (1.09-1.18)	1.09 (1.06-1.13)	1.11 (1.09-1.14)
Hispanic population[d]	0.96 (0.92-0.99)	0.95 (0.91-0.98)	0.95 (0.92-0.98)
High school graduation rate[e]	0.99 (0.94-1.05)	0.97 (0.94-1.01)	0.99 (0.95-1.02)
Median household income[f]	0.98 (0.95-1.02)	0.95 (0.92-0.98)	0.95 (0.92-0.98)

Abbreviations: aIRR, adjusted incidence rate ratio; CI, confidence interval; N, number of state-years in the analysis; N/A, not applicable.
[a] For all revenue variables, the aIRR shown applies to a $1000 increase in revenue.
[b] For the Kakwani index sensitivity analysis, the Kakwani index was the tax progressivity measure. For all other sensitivity analyses, the Suits index was the tax progressivity measure. For both indices, the aIRR shown applies to a 0.10-unit increase in the index.
[c] Year dummy variables are not shown.
[d] For non-Hispanic Black population, the aIRR shown applies to a 10% increase in percentage of the population that is non-Hispanic Black. For Hispanic population, the aIRR shown applies to a 10% increase in percentage of the population that is Hispanic.
[e] For high school graduation rate, the aIRR shown applies to a 5% increase in graduation rate.
[f] For median household income, the aIRR shown applies to a $10 000 increase in median household income.

© 2023 Junior JA et al. *JAMA Network Open.*

eTable 5. Post Hoc Sensitivity Analyses of the Association Between Tax Policy and Infant Mortality

A.

	Infant mortality aIRR (95% CI)			
	**Fixed effects (N = 300)**	**Democrat legislature percentage (N = 250)**	**Democrat legislature majority (N = 300)**	**Democrat presidential votes (N = 300)**
**Independent variables**				
Tax revenue per capita[a]	0.98 (0.96-0.99)	0.97 (0.95-0.99)	0.97 (0.95-0.99)	0.97 (0.95-0.99)
Suits index of tax progressivity[b]	0.95 (0.91-0.99)	0.95 (0.91-0.99)	0.95 (0.91-0.99)	0.95 (0.91-0.99)
**Covariates[c]**				
Federal transfer revenue per capita[a]	0.97 (0.93-1.01)	0.97 (0.93-1.00)	0.97 (0.93-1.00)	0.96 (0.93-1.00)
Other revenue per capita[a]	1.00 (0.99-1.01)	1.02 (1.01-1.03)	1.02 (1.01-1.03)	1.02 (1.01-1.03)
Non-Hispanic Black population[d]	1.11 (0.97-1.27)	1.11 (1.08-1.14)	1.11 (1.08-1.14)	1.11 (1.08-1.14)
Hispanic population[d]	0.95 (0.87-1.03)	0.95 (0.92-0.98)	0.95 (0.92-0.98)	0.95 (0.92-0.98)
High school graduation rate[e]	1.02 (0.98-1.06)	0.98 (0.94-1.02)	0.99 (0.95-1.02)	0.98 (0.95-1.02)
Median household income[f]	0.99 (0.97-1.02)	0.95 (0.92-0.98)	0.95 (0.92-0.98)	0.95 (0.92-0.99)
Democrat legislature percentage[g]	N/A	1.00 (1.00-1.00)	N/A	N/A
Democrat legislature majority[h]	N/A	N/A	1.00 (0.97-1.03)	N/A
Democrat presidential votes[i]	N/A	N/A	N/A	1.00 (1.00-1.00)

Abbreviations: aIRR, adjusted incidence rate ratio; CI, confidence interval; N, number of state-years in the analysis; N/A, not applicable.
[a] For all revenue variables, the aIRR shown applies to a $1000 increase in revenue.
[b] For the Suits index of tax progressivity, the aIRR shown applies to a 0.10-unit increase in the index.
[c] Year dummy variables are not shown.
[d] For non-Hispanic Black population, the aIRR shown applies to a 10% increase in percentage of the population that is non-Hispanic Black. For Hispanic population, the aIRR shown applies to a 10% increase in percentage of the population that is Hispanic.
[e] For high school graduation rate, the aIRR shown applies to a 5% increase in graduation rate.
[f] For median household income, the aIRR shown applies to a $10 000 increase in median household income.
[g] The aIRR shown applies to a 1% increase in the percentage of state legislators who were Democrat.
[h] The aIRR shown applies to a Democrat-majority state legislature (as compared to a Republican-majority).
[i] The aIRR shown applies to a 1% increase in the percentage of US presidential election votes cast for the Democratic Party candidate.

B

	Infant mortality aIRR (95% CI)		
	**Non-resident infant deaths (N = 300)**	**19 Vermont infant deaths in 2019 (N = 300)**	**20 Vermont infant deaths in 2019 (N = 300)**
**Independent variables**			
Tax revenue per capita[a]	0.97 (0.95-0.99)	0.98 (0.95-0.99)	0.98 (0.95-0.99)
Suits index of tax progressivity[b]	0.95 (0.91-0.99)	0.96 (0.92-0.99)	0.96 (0.92-1.00)
**Covariates[c]**			
Federal transfer revenue per capita[a]	0.97 (0.93-1.00)	0.97 (0.94-1.01)	0.97 (0.94-1.01)
Other revenue per capita[a]	1.02 (1.01-1.03)	1.01 (1.00-1.03)	1.01 (1.00-1.02)
Non-Hispanic Black population[d]	1.11 (1.08-1.14)	1.11 (1.08-1.13)	1.11 (1.08-1.13)
Hispanic population[d]	0.95 (0.92-0.98)	0.95 (0.92-0.98)	0.95 (0.92-0.98)

© 2023 Junior JA et al. *JAMA Network Open.*

High school graduation rate[e]	0.99 (0.95-1.02)	0.99 (0.95-1.02)	0.99 (0.96-1.02)
Median household income[f]	0.95 (0.92-0.98)	0.95 (0.92-0.98)	0.95 (0.92-0.98)
Non-resident infant deaths[g]	1.00 (1.00-1.00)	N/A	N/A

Abbreviations: aIRR, adjusted incidence rate ratio; CI, confidence interval; N, number of state-years in the analysis; N/A, not applicable.

[a] For all revenue variables, the aIRR shown applies to a $1000 increase in revenue.

[b] For the Suits index of tax progressivity, the aIRR shown applies to a 0.10-unit increase in the index.

[c] Year dummy variables are not shown.

[d] For non-Hispanic Black population, the aIRR shown applies to a 10% increase in percentage of the population that is non-Hispanic Black. For Hispanic population, the aIRR shown applies to a 10% increase in percentage of the population that is Hispanic.

[e] For high school graduation rate, the aIRR shown applies to a 5% increase in graduation rate.

[f] For median household income, the aIRR shown applies to a $10 000 increase in median household income.

[g] The aIRR shown applies to a 1% increase in the percentage of infant deaths comprised of out-of-state residents.

© 2023 Junior JA et al. *JAMA Network Open.*

eTable 6. Association Between the Suits Index Of Tax Progressivity and Infant Mortality at Different Levels of Tax Revenue Per Capita

Tax revenue per capita, $	Expected change in infant mortality rate (95% CI) associated with an increase of 0.10 in the Suits index of tax progressivity
2624	0.16 (-0.25 to 0.58)
3746	-0.03 (-0.37 to 0.31)
4275	-0.12 (-0.44 to 0.20)
5121	-0.26 (-0.58 to 0.06)
5550	-0.33 (-0.66 to -0.01)
13200	-1.46 (-2.55 to -0.37)

Abbreviation: CI, confidence interval.
Covariates are not shown, but all covariates are controlled for in this analysis.

© 2023 Junior JA et al. *JAMA Network Open.*

eReferences

1. Almond D, Hoynes HW, Schanzenbach DW. Inside the war on poverty: the impact of food stamps on birth outcomes. *Rev Econ Stat*. 2011;93(2):387-403. doi:10.1162/REST_a_00089
2. Arno PS, Sohler N, Viola D, Schechter C. Bringing health and social policy together: the case of the earned income tax credit. *J Public Health Policy*. 2009;30(2):198-207. doi:10.1057/jphp.2009.3
3. Ehrenthal DB, Daphne Kuo HH, Kirby RS. Infant mortality in rural and nonrural counties in the United States. *Pediatrics*. 2020;146(5). doi:10.1542/peds.2020-0464
4. Goldstein ND, Palumbo AJ, Bellamy SL, Purtle J, Locke R. State and local government expenditures and infant mortality in the United States. *Pediatrics*. 2020;146(5):e20201134. doi:10.1542/peds.2020-1134
5. Granruth LB, Shields JJ. Impact of the level of state tax code progressivity on children’s health outcomes. *Heal Soc Work*. 2011;36(3):207-215. doi:10.1093/hsw/36.3.207
6. Hirai AH, Sappenfield WM, Kogan MD, et al. Contributors to excess infant mortality in the U.S. South. *Am J Prev Med*. 2014;46(3):219-227. doi:10.1016/j.amepre.2013.12.006
7. Homan P. Political gender inequality and infant mortality in the United States, 1990–2012. *Soc Sci Med*. 2017;182:127-135. doi:10.1016/j.socscimed.2017.04.024
8. Kawachi I, Kennedy BP, Gupta V, Prothrow-Stith D. Women’s status and the health of women and men: a view from the States. *Soc Sci Med*. 1999;48(1):21-32. doi:10.1016/S0277-9536(98)00286-X
9. Kershenbaum A, Price J, Nagle NN, Campbell Erwin P. The pattern of association between U.S. economic indicators and infant mortality rates at the state level. *J Health Care Poor Underserved*. 2014;25(3):1432-1448. doi:10.1353/hpu.2014.0144
10. Kim A-S, Jennings ET. Effects of U.S. states’ social welfare systems on population health. *Policy Stud J*. 2009;37(4):745-767. doi:10.1111/j.1541-0072.2009.00333.x
11. Kim D, Saada A. The social determinants of infant mortality and birth outcomes in Western developed nations: a cross-country systematic review. *Int J Environ Res Public Health*. 2013;10(6):2296-2335. doi:10.3390/ijerph10062296
12. Komro KA, Livingston MD, Markowitz S, Wagenaar AC. The effect of an increased minimum wage on infant mortality and birth weight. *Am J Public Health*. 2016;106(8):1514-1516. doi:10.2105/AJPH.2016.303268
13. McLaughlin M, Rank MR. Impact of federal transfers upon US infant mortality rates: a secondary analysis using a fixed effects regression approach. *BMJ Open*. 2018;8:21533. doi:10.1136/bmjopen-2018-021533
14. Mehra R, Boyd LM, Ickovics JR. Racial residential segregation and adverse birth outcomes: a systematic review and meta-analysis. *Soc Sci Med*. 2017;191:237-250. doi:10.1016/j.socscimed.2017.09.018
15. Pabayo R, Cook DM, Harling G, Gunawan A, Rosenquist NA, Muennig P. State-level income inequality and mortality among infants born in the United States

© 2023 Junior JA et al. *JAMA Network Open.*

2007-2010: a cohort study. *BMC Public Health*. 2019;19(1):1333. doi:10.1186/s12889-019-7651-y
16. Pabayo R, Ehntholt A, Davis K, Liu SY, Muennig P, Cook D. Structural racism and odds for infant mortality among infants born in the United States 2010. *J Racial Ethn Heal Disparities*. 2019;6(6):1095-1106. doi:10.1007/s40615-019-00612-w
17. Reeves A, Gourtsoyannis Y, Basu S, McCoy D, McKee M, Stuckler D. Financing universal health coverage - effects of alternative tax structures on public health systems: cross-national modelling in 89 low-income and middle-income countries. *Lancet*. 2015;386(9990):274-280. doi:10.1016/S0140-6736(15)60574-8
18. Reno R, Hyder A. The evidence base for social determinants of health as risk factors for infant mortality: a systematic scoping review. *J Health Care Poor Underserved*. 2018;29(4):1188-1208. doi:10.1353/hpu.2018.0091
19. Rigby E, Hatch ME. Incorporating economic policy into a “health-in-all-policies” agenda. *Health Aff*. 2016;35(11):2044-2052. doi:10.1377/hlthaff.2016.0710
20. Rodriguez JM, Bound J, Geronimus AT. US infant mortality and the President’s party. *Int J Epidemiol*. 2014;43(3):818-826. doi:10.1093/ije/dyt252
21. Rodriguez JM. The politics hypothesis and racial disparities in infants’ health in the United States. *SSM - Popul Heal*. 2019;8:100440. doi:10.1016/j.ssmph.2019.100440
22. Ruiz JI, Nuhu K, McDaniel JT, Popoff F, Izcovich A, Criniti JM. Inequality as a powerful predictor of infant and maternal mortality around the world. Carpenter DO, ed. *PLoS One*. 2015;10(10):e0140796. doi:10.1371/journal.pone.0140796
23. Siddiqi A, Jones MK, Campbell Erwin P. Does higher income inequality adversely influence infant mortality rates? Reconciling descriptive patterns and recent research findings. *Soc Sci Med*. 2015;131:82-88. doi:10.1016/j.socscimed.2015.03.010
24. Siddiqi A, Jones MK, Bruce DJ, Erwin PC. Do racial inequities in infant mortality correspond to variations in societal conditions? A study of state-level income inequality in the U.S., 1992-2007. *Soc Sci Med*. 2016;164:49-58. doi:10.1016/j.socscimed.2016.07.013
25. Soneji S, Beltrán-Sánchez H. Association of Special Supplemental Nutrition Program for Women, Infants, and Children with preterm birth and infant mortality. *JAMA Netw Open*. 2019;2(12):e1916722. doi:10.1001/jamanetworkopen.2019.16722
26. Wallace M, Crear-Perry J, Richardson L, Tarver M, Theall K. Separate and unequal: structural racism and infant mortality in the US. *Health Place*. 2017;45:140-144. doi:10.1016/j.healthplace.2017.03.012
27. Chen A, Oster E, Williams H. Why is infant mortality higher in the United States than in Europe? *Am Econ J Econ Policy*. 2016;8(2):89-124. doi:10.1257/pol.20140224
28. Ehntholt A, Cook DM, Rosenquist NA, Muennig P, Pabayo R. State- and county-level income inequality and infant mortality in the USA in 2010: a cohort study. *Int J Public Health*. 2020;65(6):769-780. doi:10.1007/s00038-020-01388-1
29. Babyak MA. What you see may not be what you get: a brief, nontechnical introduction to overfitting in regression-type models. *Psychosom Med*. 2004;66(3):411-421. Accessed January 30, 2021.

© 2023 Junior JA et al. *JAMA Network Open.*

https://pubmed.ncbi.nlm.nih.gov/15184705/
30. Hilbe JM. *Modeling Count Data*. Cambridge University Press; 2014. doi:10.1017/CBO9781139236065
31. Babyak MA. Understanding confounding and mediation. *Evid Based Ment Health*. 2009;12(3):68-71. doi:10.1136/ebmh.12.3.68
32. Wang X, Cheng Z. Cross-sectional studies: strengths, weaknesses, and recommendations. *Chest*. 2020;158(1):S65-S71. doi:10.1016/J.CHEST.2020.03.012
33. Lhamon CE, Heriot G, Kirsanow PN, et al. *Targeted Fines and Fees Against Communities of Color: Civil Rights & Constitutional Implications*. U.S. Commission on Civil Rights; 2017. Accessed February 10, 2021. https://www.usccr.gov/pubs/2017/Statutory_Enforcement_Report2017.pdf
34. Shoub K, Christiani L, Baumgartner FR, Epp DA, Roach K. Fines, Fees, Forfeitures, and Disparities: A Link Between Municipal Reliance on Fines and Racial Disparities in Policing. *Policy Stud J*. Published online August 7, 2020:psj.12412. doi:10.1111/psj.12412
35. Davis BA, Arcaya MC, Williams DR, Krieger N. The impact of county-level fees & fines as exploitative revenue generation on US birth outcomes 2011–2015. *Heal Place* . 2023;80:102990. Accessed March 5, 2023. https://doi.org/10.1016/j.healthplace.2023.102990
36. Kamal R, Hudman J, McDermott D. *What Do We Know about Infant Mortality in the U.S. and Comparable Countries?* Peterson-KFF; 2019. Accessed January 17, 2021. https://www.healthsystemtracker.org/chart-collection/infant-mortality-u-s-compare-countries/#item-the-u-s-infant-mortality-rate-has-improved-over-time
37. O’Brien RL. Redistribution and the new fiscal sociology: race and the progressivity of state and local taxes. *Am J Sociol*. 2017;122(4):1015-1049. doi:10.1086/690118
38. Newman KS, O’Brien R. *Taxing the Poor: Doing Damage to the Truly Disadvantaged*. University of California Press; 2011. Accessed January 21, 2021. https://www.ucpress.edu/book/9780520269675/taxing-the-poor
39. Marshall J. The anti-Democrat diploma: how high school education decreases support for the Democratic Party. *Am J Pol Sci*. 2019;63(1):67-83. doi:10.1111/ajps.12409
40. Bullock JG. Education and attitudes toward redistribution in the United States. *Br J Polit Sci*. Published online 2020:1-21. doi:10.1017/S0007123419000504
41. Scheve K, Stasavage D. *Taxing the Rich: A History of Fiscal Fairness in the United States and Europe*. Princeton University Press; 2016. Accessed February 6, 2021. https://press.princeton.edu/books/hardcover/9780691165455/taxing-the-rich
42. Fitzmaurice GM, Laird NM, Ware JH. Fixed effects versus random effects models. In: *Applied Longitudinal Analysis*. John Wiley & Sons, Inc; 2011:241-264. Accessed November 15, 2021. https://doi.org/10.1002/9781119513469
43. Schempf AH, Kaufman JS. Accounting for context in studies of health inequalities: a review and comparison of analytic approaches. *Ann Epidemiol*. 2012;22(10):683-690. doi:10.1016/J.ANNEPIDEM.2012.06.105
44. Hilbe JM. Count panel models. In: *Negative Binomial Regression*. Cambridge

© 2023 Junior JA et al. *JAMA Network Open.*

University Press; 2011:447-501. doi:10.1017/CBO9780511973420.015
45. Hubbard AE, Ahern J, Fleischer NL, et al. To GEE or not to GEE: comparing population average and mixed models for estimating the associations between neighborhood risk factors and health. *Epidemiology*. 2010;21(4):467-474. doi:10.1097/EDE.0B013E3181CAEB90
46. French B, Stuart EA. Study designs and statistical methods for studies of child and adolescent health policies. *JAMA Pediatr*. 2020;174(10):925-927. doi:10.1001/JAMAPEDIATRICS.2020.3408
47. The Council of State Governments. The Book of the States Archive. Accessed February 16, 2023. https://issuu.com/csg.publications/stacks/46495f12f95847e6935d331969ed650a
48. Massachusetts Institute of Technology (MIT) Election Data and Science Lab. U.S. President 1976–2020. doi:10.7910/DVN/42MVDX
49. United States Department of Health and Human Services, Centers of Disease Control and Prevention, National Center for Health Statistics, Division of Vital Statistics. CDC WONDER Online Database: Linked Birth / Infant Death Records. Accessed February 13, 2021. https://wonder.cdc.gov/lbd.html
50. Bell ML, Belanger K. Review of research on residential mobility during pregnancy: consequences for assessment of prenatal environmental exposures. *J Expo Sci Environ Epidemiol*. 2012;22(5):429-438. doi:10.1038/JES.2012.42
51. United States Department of Health and Human Services Centers of Disease Control and Prevention, National Center for Health Statistics, Division of Vital Statistics. Infant Deaths Linked Birth / Infant Death Records Data Summary. Accessed February 21, 2023. https://wonder.cdc.gov/wonder/help/lbd.html

© 2023 Junior JA et al. *JAMA Network Open.*
