# Supplementary material for: Association of State-Level Tax Policy and Infant Mortality in the United States, 1996-2019
Source: JAMA Netw Open. 2023 Apr 24;6(4):e239646. doi: 10.1001/jamanetworkopen.2023.9646 (PMC10126872; doi:10.1001/jamanetworkopen.2023.9646)
Supplement: Supplement 2. — Data Sharing Statement [file jamanetwopen-e239646-s002.pdf]

Data Sharing Statement

Junior. Association of State-Level Tax Policy and Infant Mortality in the United States, 1996-2019. *JAMA Netw Open*. Published April 24, 2023. doi:10.1001/jamanetworkopen.2023.9646

Data
Data available: No

Additional Information
Explanation for why data not available: All data used are from publicly available sources. So there is not a need for us to supply this data. However, we will make the dataset we compiled of independent variables and covariates available upon request.
